# Plasma renin levels are associated with cardiac function in primary adrenal insufficiency

**DOI:** 10.1007/s12020-019-01974-1

**Published:** 2019-06-08

**Authors:** Peter Wolf, Hannes Beiglböck, Paul Fellinger, Lorenz Pfleger, Stefan Aschauer, Alois Gessl, Rodrig Marculescu, Siegfried Trattnig, Alexandra Kautzky-Willer, Anton Luger, Yvonne Winhofer, Martin Krššák, Michael Krebs

**Affiliations:** 1 Division of Endocrinology and Metabolism, Department of Internal Medicine III, Medical University of Vienna, Vienna, Austria; 2 Centre of Excellence - High Field MR, Department of Biomedical Imaging and Image-guided Therapy, Medical University of Vienna, Vienna, Austria; 3 Division of Cardiology, Department of Internal Medicine II, Medical University of Vienna, Vienna, Austria; 4 Department of Laboratory Medicine, Medical University of Vienna, Vienna, Austria

**Keywords:** Ectopic lipids, Addison’s disease, Glucocorticoid replacement therapy, Renin–angiotensin–aldosterone system

## Abstract

**Background:**

Despite adequate glucocorticoid (GC) and mineralocorticoid (MC) replacement therapy, primary adrenal insufficiency (AI) is associated with an increased mortality, mainly due to cardiovascular disease. The role of MC replacement is not known. Therefore, we assessed whether renin concentrations during routine GC and MC substitution therapy are associated with heart function and morphology.

**Methods:**

Thirty two patients with primary AI were included in a cross-sectional case–control study. In total, 17 patients and 34 healthy controls (age: 48 ± 12 vs. 46 ± 18 years; BMI: 23 ± 3 vs. 24 ± 3 kg/m^2^) underwent magnetic resonance spectroscopy and imaging measurements to assess cardiac function, morphology, ectopic lipids, and visceral/subcutaneous fat mass. Patients were divided according to their actual plasma renin concentration at the study visit (Actual-Renin_low_ vs. Actual-Renin_high_) and their median plasma renin concentration of previous visits (Median-Renin_low_ vs. Median-Renin_high_).

**Results:**

Ejection fraction was higher (67 ± 5 vs. 55 ± 3%; *p* = 0.001) and left ventricular mass was lower (60 ± 9 vs. 73 ± 10 g/m^2^; *p* = 0.025) in Actual-Renin_high_. Median-Renin_high_ was associated with lower cardiac mass (64 ± 9 vs. 76 ± 11 g/m^2^; *p* = 0.029). Blood pressure, glucose, and lipid metabolism, as well as ectopic lipid content, pericardial fat mass, and visceral/subcutaneous fat were not different between the groups. Compared with controls, ejection fraction was significantly lower in patients with AI (56 ± 4 vs. 63 ± 8%; *p* = 0.019). No differences were found in patients with ≤20 mg compared with >20 mg of hydrocortisone per day.

**Conclusions:**

Higher renin concentrations are associated with more favorable cardiac function and morphology in patients with primary AI.

## Background

Primary adrenal insufficiency (AI) is characterized by potentially life-threatening glucocorticoid (GC) and mineralocorticoid (MC) deficiency, requiring lifelong hormone replacement therapy [1]. In contrast to reports of retrospective cohort studies indicating that regular GC and MC substitution therapy might normalize life expectancy, with excess mortality only in patients diagnosed at a younger age [2], other evidence hints at a more than twofold increase in mortality in affected patients compared with the general population, mainly due to increased risk of cardiovascular disease [3, 4].

There are several explanations addressing cardiovascular risk factors, which mainly focus on GC replacement therapy. For example, even a small oversupply of daily GC dose is associated with an adverse cardiovascular risk profile, including an increase in waist circumference, blood pressure, and fasting glucose [5]. Also, the timing of GC replacement therapy might be of importance. In contrast to conventional therapy, when GC is administered two to three times daily to mimic the physiological cortisol rhythm, studies investigating modified-release preparations of hydrocortisone report significant improvements in body mass index (BMI), glucose, and lipid metabolism [6, 7].

Only little is known about the role of MC replacement therapy in the development of cardiovascular diseases in patients suffering from primary AI. The most important hormone in MC metabolism is aldosterone, which is mainly regulated by the renin–angiotensin–aldosterone (RAS) system. Excessive aldosterone secretion is well known to be associated with an increased mortality, due to myocardial infarction, stroke, or heart failure [8, 9]. On the other hand, RAS consists of an enzymatic cascade of various angiotensin metabolites, which also exert effects on cardiovascular risk factors. The most prominent RAS metabolite is angiotensin 2 (AT2), which promotes vasoconstriction, cardiac remodeling, and modulates systemic inflammatory response to vascular injury similar to aldosterone [10, 11]. In contrast, other angiotensin metabolites like Angiotensin 1–7 (Ang1–7) are potentially cardioprotective and might counterbalance the adverse effects of AT2 [12]. Preliminary data of our study group indicate that the RAS is highly upregulated with increased concentrations of AT2 and Ang1–7 in patients suffering from primary AI, despite adequate GC and MC replacement therapy [13]. In addition, plasma renin concentration strongly correlates with RAS activity and AT2 [13].

Clinical practice guidelines recommend titration of MC replacement therapy to aim a plasma renin concentration in the upper reference range [14]. However, clinical evidence supporting this recommendation is scarce and the role of MC in the development of cardiovascular disease in patients with primary AI is unknown. Concentrations of both aldosterone and AT2 are closely correlated with plasma renin levels. Therefore, we aimed to investigate the impact of plasma renin concentration on cardiovascular risk factors in patients with stable hormone replacement therapy.

## Methods

We performed a cross-sectional, single-center, pilot study at the Medical University of Vienna, Department of Internal Medicine III, Division of Endocrinology and Metabolism. The study protocol was approved by the local ethics committee and written informed consent was obtained from all participating subjects. This study was conducted in conformance with the relevant guidelines and regulations, i.e., principles of the Declaration of Helsinki and the ICH-GCP guidelines.

**Fig. 1:**
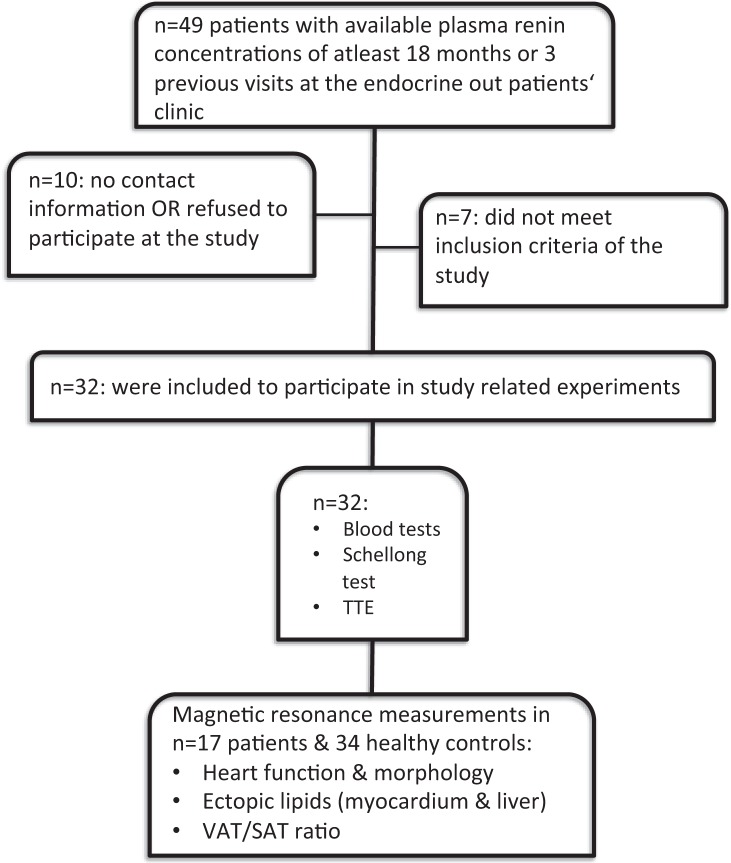
Flowchart: recruitment process of all patients with available plasma renin concentrations of previous visits at the endocrine outpatients’ clinic. TTE transthoracic echocardiography, VAT visceral adipose tissue, SAT subcutaneous adipose tissue

Patients with confirmed primary AI treated at the endocrine outpatients’ clinic at the Medical University of Vienna were searched for available plasma renin concentrations of previous visits. Included patients had to be on stable GC and MC replacement therapy for at least 6 months prior to the study visit. Exclusion criteria were concomitant antihypertensive therapy with ACE inhibitors/AT2 antagonists, diabetes mellitus (type 1 and 2), elevated liver enzymes > 3 × upper limit of normal, medical history of cardiovascular disease, and advanced chronic kidney disease (estimated glomerular filtration rate < 45 ml/min). In total, 32 patients with primary adrenal insufficiency could be included (see Fig. 1). In all patients, primary AI was confirmed at initial diagnosis by morning cortisol < 5 μg/dl, together with ACTH > 2-fold the upper limit of normal, according to current guidelines [14]. The cause of primary AI was autoimmune adrenalitis in most of the patients, confirmed by positive testing of 21-hydroxylase antibodies. Two patients suffered from adrenoleukodystrophy. In all patients, GC replacement therapy was performed with standard rapid-release hydrocortisone. Twenty nine patients received daily MC replacement therapy.

All patients underwent clinical examination and blood tests, as well as the assessment of anthropometric characteristics, a Schellong test, and a transthoracic echocardiography.

Seventeen patients volunteered to participate at magnetic resonance (MR) spectroscopy and imaging measurements at a second study day. These patients were compared with 34 healthy control subjects matched for age and body mass index (BMI) in a 1:2 ratio.

In order to compare RAS activity, patients were divided into two groups of similar sample size, according to their actual plasma renin concentration (Actual-Renin_low_: <50 μIU/ml and Actual-Renin_high_: >50 μIU/ml), as well as to their median plasma renin concentration of previous visits at the endocrine outpatients’ clinic (Median-Renin_low_: <100 μIU/ml and Median-Renin_high_: >100 μIU/ml). The normal range in supine position of the renin assay used was 4.4–46.1 μIU/ml with a CV of 6.6%. Actual-Renin_high_ reflects about >onefold upper-normal range and Median-Renin_high_ about >twofold upper-normal range. Different cutoff values for actual-Renin and Median-Renin had to be chosen to obtain two groups of comparable sample size. The mean time between the first and last of these previous visits was 31 ± 16 months.

In total, 87.5% of all included patients stayed within their groups, i.e., Actual-Renin_low _= Median-Renin_low_/Actual-Renin_high_ = Median-Renin_high_. Two patients in the Actual-Renin_low_ group switched to the Median-Renin_high_ group and vice versa.

To evaluate the impact of daily glucocorticoid replacement therapy, patients were divided into daily hydrocortisone dose of ≤20 mg/day and daily hydrocortisone dose of >20 mg/day.

Blood tests were performed under standardized conditions in a seated position, following at least 15 min of physical rest. Laboratory parameters were measured by routine lab methods at the department of laboratory medicine of the Medical University of Vienna (http://www.kimcl.at).

All transthoracic echocardiography (TTE) measurements were performed using commercially available equipment (Vivid 7 aGE Healthcare, Chicago, IL) by the same experienced physician according to current guidelines [15].

To evaluate postural hypotension, Schellong test was performed in all patients. Therefore, blood pressure was measured every 2 min for 10 min in supine position and immediately, as well as every 2 min for 10 min after promptly getting up.

All magnetic resonance spectroscopy and imaging measurements were performed on a 3.0-T Tim Trio System (Siemens Healthcare, Erlangen, Germany) operated with a Syngo VB17 (Siemens Healthcare) user interface after an overnight fast. Myocardial lipid content (MYCL) was measured by ECG-gated single-voxel-localized ^1^H-MRS. The volume of interest was positioned in the interventricular septum to avoid signal alterations by the epicardial fat. PRESS sequence (typical VOI = 15 × 10 × 30 mm^3^; TE = 30 ms; NA = 2 × 4 for water signal; 2 × 8 for water-suppressed signal) data acquisition was performed during multiple single-breath holds. MYCL was calculated from the ratio of the summed area of methylene and methyl groups to that of water, following the individual spin–spin relaxation correction as the percentage of tissue water MRS signal. For cardiac MR visualization and analyses, ARGUS software (Siemens AG Healthcare, Erlangen, Germany) was used. Left ventricular function by MR imaging was assessed manually and has also been described in detail previously [16–18]. As published in ref. [18], pericardial fat (PERI), i.e., paracardial + epicardial adipose tissue, regions of interest were manually drawn along the borders of the fat surrounding the heart in three slices from the apex to the pulmonary trunk. The mean is given in cm^2^. Quantitation of hepatocellular lipid content (HCL) was performed using localized single-voxel ^1^H-MRS like that published previously [16–18]. PRESS sequence (VOI = 3 × 3 × 3 cm^3^; TE = 30 ms; NA = 4 for each TE) data acquisition was performed during single-breath hold. HCL was calculated from the ratio of the summed area of methylene and methyl lipid signals to that of water following the individual spin–spin relaxation correction as the percentage of tissue total MRS signal. The amount of visceral and subcutaneous adipose tissue (VAT, SAT) was assessed from T_1_-weighted axial 2D turbo spin-echo multislice images (TE = 40 ms, TR = 450 ms, Matrix 256 × 144, and FOV adjusted to the body size) over the abdominal region within single-breath hold. The signal of adipose tissue within the image slice in the height of L4–L5 was semiautomatically segmented off-line within Jim 5.0 Image Analysis software (Xinapse Systems Ltd, West Bergholt, UK) and the amount of VAT and SAT was given as area within the slice in cm^2^ [16].

Based on our recent data in nondiabetic individuals [16], a sample size of eight patients per group was sufficient to observe a significant difference in the primary outcome parameter left ventricular myocardial mass with alpha = 0.05 and beta = 0.2 between the groups.

Exploratory statistical analysis was performed using SPSS Version 24 (IBM, Armonk, NY, USA). Normal distribution was checked by data visualization. Data are given as means ± standard deviation or as median (minimum; maximum). Comparison between groups was performed by unpaired Student’s *t* tests or Mann–Whitney *U* tests. Nominal parameters were compared by chi-square tests. Analysis of variances was calculated with ANOVA. Correlation analysis was calculated with Spearman’s correlation coefficient (r). Due to the hypothesis-generating design of the study, data were not corrected for multiple testing. The level of statistical significance was set at *α* < 0.05.

## Results

Thirty two patients (women/men: 21/11; age: 55 ± 17 years; BMI: 25 ± 5 kg/m^2^; disease duration: 19 ± 10 years) were included in this study. All patients were on stable GC replacement therapy (mean GC dose: 23 ± 5 mg of hydrocortone/day) and 30 patients were on stable MC replacement therapy (mean MC dose: 0.06 ± 0.03 mg of fludrocortisone/day). In nine patients, daily GC dose exceeded 20 mg of hydrocortisone per day.

TTE showed normal left ventricular (ejection fraction: 57 ± 5%; average global longitudinal strain: −19 ± 2%) and right ventricular (TAPSE: 26 ± 4 mm) systolic function in all investigated patients. Grade I diastolic dysfunction, i.e., impaired diastolic relaxation, was present in 13 patients (43%). One patient (3%) presented with grade III diastolic dysfunction.

### The impact of actual plasma renin concentration

Patients suffering from primary AI were grouped according to their actual plasma renin concentration measured at the study visit (Actual-Renin_low_ vs. Actual-Renin_high_). Anthropometric characteristics, disease duration, as well as daily dose of GC and MC replacement therapy were comparable (see Table 1).

**Table 1:** Anthropometric characteristics, blood pressure, and parameters of glucose and lipid metabolism in Actual-Renin_low_ and Actual-Renin_high_ according to actual renin concentrations at the time of the study visit; magnetic resonance (MR) measurements were performed in *n* = 9 patients in Actual-Renin_low_ and *n* = 7 patients in Actual-Renin_high_; data are given as means ± standard deviation or as median (minimum; maximum)

	Actual-Renin_low_	Actual-Renin_high_	*p* Value
Sex (f/m)	8/5	13/5	n.s.
Age (years)	53 ± 14	57 ± 14	n.s.
BMI (kg/m^2^)	25 ± 5	25 ± 5	n.s.
Disease duration (years)	20 ± 11	18 ± 9	n.s.
MC dose (mg/d)	0.06 ± 0.04	0.06 ± 0.03	n.s.
GC dose (mg/d)	23 ± 6	23 ± 5	n.s.
Plasma renin concentration (μIU/ml)	27 (2.2; 44)	169 (61; 2834)	*<*0.001*
Actual systolic RR (mmHg)	121 ± 14	133 ± 19	n.s.
Actual diastolic RR (mmHg)	72 ± 6	72 ± 11	n.s.
Triglycerides (mg/dl)	113 ± 75	134 ± 58	n.s.
Total cholesterol (mg/dl)	168 ± 61	187 ± 54	n.s.
HDL cholesterol (mg/dl)	67 ± 17	61 ± 14	n.s.
LDL cholesterol (mg/dl)	98 ± 31	111 ± 32	n.s.
Glucose (mg/dl)	82 ± 9	89 ± 10	0.053
HbA1c (%)	5.1 ± 0.2	5.4 ± 0.3	0.027*
HOMA_IR	2.0 ± 1.8	2.9 ± 1.8	n.s.
*MR imaging and spectroscopy*			
Ejection fraction (%)	55 ± 3	67 ± 5	0.001*
Left ventricular mass (g/m^2^)	73 ± 10	60 ± 9	0.025*
MYCL (%)	0.32 ± 0.2	0.43 ± 0.4	n.s.
HCL (%)	2.1 ± 1.7	2.7 ± 2.7	n.s.
Epicardial fat content (cm^2^)	14 ± 7	17 ± 8	n.s.
VAT/SAT ratio (mm^2^)	2.7 ± 1.3	2.2 ± 0.8	n.s.
*TTE*			
Global longitudinal strain (%)	−19 ± 2	−18 ± 2	n.s.
E/A ratio	1.4 ± 0.4	−0.3 ± 0.2	n.s.

*BMI* body mass index, *MC dose* daily dose of mineralocorticoid replacement therapy, *GC dose* daily dose of glucocorticoid replacement therapy, *actual systolic and diastolic RR* systolic and diastolic blood pressure at the time point of the study visit, *TTE* transthoracic echocardiography, *MYCL* intramyocardial lipid content, *HCL* hepatocellular lipid content, *VAT/SAT* visceral adipose tissue/subcutaneous adipose tissue

*p < 0.05

With regard to glucose metabolism, HbA1c was significantly lower in Actual-Renin_low_. Also, fasting blood glucose concentration tended to be lower in Actual-Renin_low_ without reaching statistical significance (see Table 1).

As assessed by MR imaging, ejection fraction was significantly higher and left ventricular mass was significantly lower in Actual-Renin_high_ compared with Actual-Renin_low_. Global longitudinal strain and diastolic function were assessed by TTE and did not differ between the groups (see Table 1).

Analysis of variance in all patients with primary AI with ejection fraction or left ventricular mass as dependent variables and actual renin concentration as an independent variable showed significant between-group differences (ejection fraction: *F* = 24.526; *p* < 0.001; left ventricular mass: *F* = 6.254; *p* = 0.025).

In order to evaluate routine clinical signs and laboratory parameters of MC replacement therapy, patients grouped in Actual-Renin_low_ versus Actual-Renin_high_ were compared.

Salt craving and postural hypotension were three times more frequent in Actual-Renin_high_. Serum sodium concentration was lower in Actual-Renin_high_, whereas potassium level was comparable (see Table 2).

**Table 2:** Clinical signs and laboratory parameters to evaluate mineralocorticoid replacement therapy in patients with actual renin concentrations at the time of the study visit <50 μIU/ml (Actual-Renin_low_) and >50 μIU/ml (Actual-Renin_high_); data are given as means ± standard deviation or as median (minimum; maximum)

	Actual-Renin_low_	Actual-Renin_high_	*p* Value
Salt craving (% of patients)	15	39	n.s.
Postural hypotension (% of patients)	33	83	0.019*
Sodium (mmol/l)	139 ± 3	137 ± 3	0.042*
Potassium (mmol/l)	4.5 ± 0.6	4.3 ± 0.4	n.s.
ACTH (pg/ml)	232 (2; 950)	353 (1; 1824)	n.s.
ADH (pmol/l)	6.4 ± 3	8.5 ± 7	n.s.
proBNP (pg/ml)	46 (10; 201)	70 (5; 241)	n.s.

*p < 0.05

### The impact of median long-term plasma renin concentration

Patients suffering from primary AI were additionally grouped according to their plasma renin concentration during previous visits (Median-Renin_low_ vs. Median-Renin_high_). Age, sex, BMI, and disease duration were comparable between the groups. Also, the average daily dose of GC and MC replacement therapy was comparable. In addition, systolic and diastolic blood pressure at the time of the study visit, as well as mean blood pressure of previous visits was similar in patients with high- and low-median renin concentrations.

Similar to the comparison of actual renin concentrations, end-diastolic left ventricular mass was significantly higher and ejection fraction tended to be lower in Median-Renin_low_ compared with Median-Renin_high_, as assessed by MR imaging.

In TTE measurements, left ventricular global longitudinal strain, as well as diastolic function was not different between the two groups (see Table 3).

**Table 3:** Anthropometric characteristics, blood pressure, and parameters of glucose and lipid metabolism in patients divided into Median-Renin_low_ and Median-Renin_high_ according to plasma renin concentrations during the previous visits; mean time between the first and last of these previous visits was 31 ± 16 months; data are given as means ± standard deviation or as median (minimum; maximum); magnetic resonance (MR) measurements were performed in *n* = 8 patients in Actual-Renin_low_ and *n* = 9 patients in Actual-Renin_high_

	Median-Renin_low_	Median-Renin_high_	*p* Value
Sex (f/m)	8/5	13/6	n.s.
Age (years)	55 ± 15	54 ± 14	n.s.
BMI (kg/m^2^)	25 ± 5	25 ± 5	n.s.
Disease duration (years)	23 ± 10	16 ± 9	n.s.
MC dose (mg/d)	0.05 ± 0.03	0.06 ± 0.03	n.s.
GC dose (mg/d)	22 ± 6	23 ± 5	n.s.
Actual systolic RR (mmHg)	123 ± 12	124 ± 18	n.s.
Actual diastolic RR (mmHg)	72 ± 7	73 ± 12	n.s.
Mean previous systolic RR (mmHg)	131 ± 16	122 ± 16	n.s.
Mean previous diastolic RR (mmHg)	81 ± 7	80 ± 8	n.s.
Median renin concentration (μIU/ml)	46 (4; 96)	180 (101; 1501)	*<*0.001*
Actual renin concentration (μIU/ml)	31 (2; 194)	149 (25; 2834)	*<*0.001*
Triglycerides (mg/dl)	117 ± 77	134 ± 54	n.s.
Total cholesterol (mg/dl)	171 ± 64	187 ± 52	n.s.
HDL cholesterol (mg/dl)	62 ± 17	62 ± 15	n.s.
LDL cholesterol (mg/dl)	106 ± 40	109 ± 27	n.s.
Glucose (mg/dl)	86 ± 10	85 ± 10	n.s.
HbA1c (%)	5.1 ± 0.3	5.3 ± 0.3	n.s.
HOMA_IR	2.6 ± 2.3	2.5 ± 1.2	n.s.
*MR imaging and spectroscopy*			
Ejection fraction (%)	56 ± 4	63 ± 8	0.073
Left ventricular mass (g/m^2^)	76 ± 11	64 ± 9	0.029*
MYCL (%)	0.29 ± 0.2	0.43 ± 0.4	n.s.
HCL (%)	1.8 ± 1.7	2.9 ± 2.3	n.s.
Epicardial fat content (cm^2^)	16 ± 7	15 ± 8	n.s.
VAT/SAT ratio (mm^2^)	2.6 ± 1.4	2.3 ± 0.9	n.s.
*TTE*			
Global longitudinal strain (%)	−19 ± 2	−19 ± 2	n.s.
E/A ratio	1.3 ± 0.8	1.1 ± 0.4	n.s.

*BMI* body mass index, *MC dose* daily dose of mineralocorticoid replacement therapy, *GC dose* daily dose of glucocorticoid replacement therapy, *actual systolic and diastolic RR* systolic and diastolic blood pressure at the time point of the study visit, *mean previous systolic and diastolic RR* average systolic and diastolic blood pressure during the previous four routine clinical visits at the endocrinology outpatients clinic, *TTE* transthoracic echocardiography, *MYCL* intramyocardial lipid content, *HCL* hepatocellular lipid content, *VAT/SAT* visceral adipose tissue/subcutaneous adipose tissue

*p < 0.05

In correlation analysis, actual plasma renin concentration at the time of the study visit strongly correlated with left ventricular ejection fraction (*r* = 0.741; *p* < 0.001). With regard to median renin concentration, a positive association with left ventricular ejection fraction (*r* = 0.435; *p* = 0.081) and a negative association with end-diastolic septal thickness (*r* = –0.429; *p* = 0.086) could be found, yet both not reaching statistical significance.

### The impact of daily hydrocortisone dose

No difference in glucose (glucose: 87 ± 11 vs. 85 ± 10 mg/dl; *p* = n.s.; HbA1c: 5.4 ± 0.3 vs. 5.2 ± 0.3; *p* = n.s.; HOMA_IR: 2.8 ± 1.4 vs. 2.4 ± 1.8; p = n.s.) and lipid (triglycerides: 137 ± 70 vs. 123 ± 65 mg/dl; *p* = n.s.; total cholesterol: 184 ± 75 vs. 180 ± 50 mg/dl; *p* = n.s.) metabolism was found in patients with >20 mg (*n* = 9) compared with patients with ≤20 mg (*n* = 23) of daily hydrocortisone.

In addition, parameters of cardiac function (ejection fraction: 59 ± 8 vs. 61 ± 7%; *p* = n.s.) and morphology (end-diastolic left ventricular mass: 66 ± 6 vs. 71 ± 13 g/m^2^; *p* = n.s.) were comparable. Also, ectopic lipid content (HCL: 3.1 ± 2.9 vs. 2.0 ± 1.5%; *p* = n.s.; MYCL: 0.31 ± 0.2 vs. 0.41 ± 0.4%; *p* = n.s.), pericardial fat mass (16 ± 9 vs. 15 ± 6 cm^2^; *p* = n.s.), and the ratio of subcutaneous to visceral adipose tissue (2.5 ± 1.3 vs. 2.4 ± 1.1 mm^2^; *p* = n.s.) was not different.

### Comparison of patients suffering from adrenal insufficiency and healthy controls

Systolic and diastolic blood pressure and lipid metabolism were similar between the groups. With regard to glucose metabolism, fasting glucose concentration was lower in AI, whereas HbA1c and HOMA_IR were comparable with controls. In addition, ectopic lipid content in the liver and heart, as well as epicardial fat mass was similar in patients with AI and controls.

**Fig. 2:**
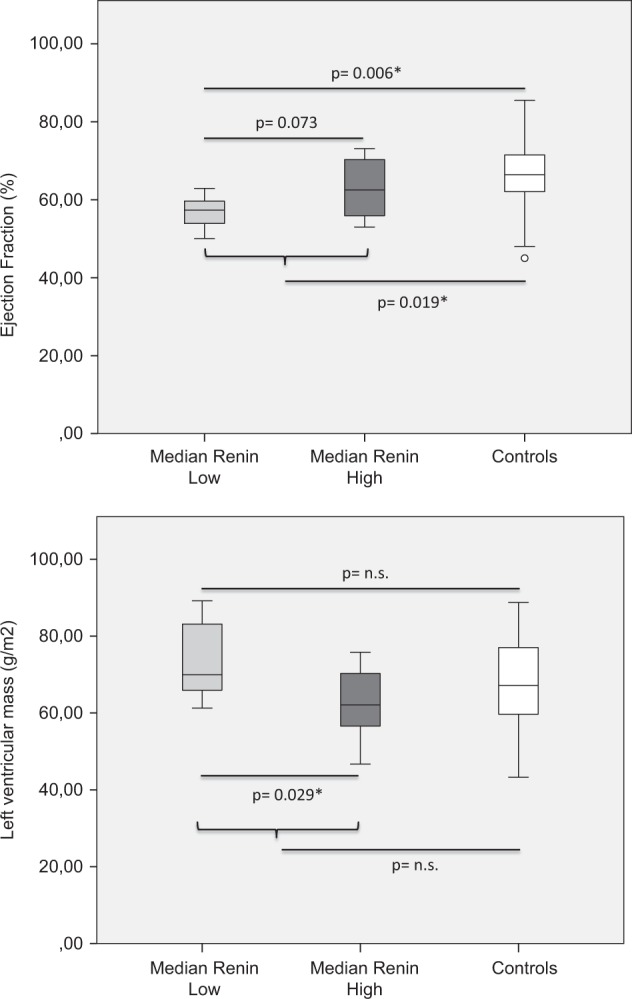
Ejection fraction and left ventricular mass in patients with normal to slightly elevated (Median Renin Low; bright gray boxplot), elevated (Median Renin High; dark gray boxplot) plasma renin concentrations during the previous four routine clinical visits at the endocrinology outpatients clinic, and healthy controls (Controls; white boxplot); comparison between different groups was performed by using unpaired Student’s *t* test; *p < 0.05

Ejection fraction was significantly lower in patients suffering from AI, whereas no differences in parameters of cardiac morphology were found. When healthy subjects were compared with patients grouped according to their plasma renin concentration of previous visits, only differences between Median-Renin_low_ and controls could be observed (see Fig. 2). For a detailed list of all parameters see Table 4.

**Table 4:** Results of *n* = 17 patients suffering from adrenal insufficiency (AI), who underwent magnetic resonance spectroscopy and imaging measurements and *n* = 34 healthy controls; anthropometric characteristics, data of laboratory tests, and results of magnetic resonance spectroscopy (MYCL intramyocardial lipid content, HCL hepatocellular lipid content) and imaging (parameters of cardiac function and morphology and epicardial fat) measurements are given as means ± standard deviation or as median (minimum; maximum); BMI body mass index

	AI	Controls	*p* Value
Age (years)	48 ± 12	46 ± 18	n.s.
BMI (kg/m^2^)	23 ± 3	24 ± 3	n.s.
Systolic RR (mmHg)	120 ± 12	129 ± 25	n.s.
Diastolic RR (mmHg)	72 ± 6	75 ± 12	n.s.
Triglycerides (mg/dl)	111 ± 62	117 ± 95	n.s.
Total cholesterol (mg/dl)	180 ± 54	192 ± 35	n.s.
HDL cholesterol (mg/dl)	61 ± 14	56 ± 13	n.s.
LDL cholesterol (mg/dl)	112 ± 28	112 ± 33	n.s.
Glucose (mg/dl)	82 ± 8	89 ± 8	0.010*
HbA1c (%)	5.3 ± 0.3	5.2 ± 0.4	n.s.
HOMA_IR	1.9 ± 1	2.4 ± 1.4	n.s.
Ejection fraction (%)	56 ± 4	63 ± 8	0.019*
End-diastolic volume (ml/m^2^)	55 ± 9	52 ± 17	n.s.
End-systolic volume (ml/m^2^)	22 ± 6	18 ± 8	n.s.
Stroke volume (ml/m^2^)	33 ± 6	33 ± 11	n.s.
Cardiac index (l/min/m^2^)	2.1 ± 0.3	2.3 ± 0.6	n.s.
End-diastolic myocardial mass (g/m^2^)	67 ± 11	67 ± 13	n.s.
End-diastolic septal thickness (mm)	8.8 ± 1.3	9 ± 1.6	n.s.
Epicardial fat (cm^2^)	16 ± 7	15 ± 10	n.s.
MYCL (%)	0.4 ± 0.3	0.4 ± 0.3	n.s.
HCL (%)	1.5 (0.3; 8.1)	3.2 (0.4; 23)	n.s.

*BMI* body mass index

*p < 0.05

## Discussion

Our data demonstrate a significant impact of RAS activity on heart function and morphology in patients suffering from primary AI. Higher renin concentrations are associated with a favorable cardiac function and morphology, i.e., an improved systolic function and a lower left ventricular mass. Interestingly, these changes were independent of conventional cardiovascular risk factors like body weight, blood pressure, glucose, and lipid metabolism, supporting the importance of direct MC action on the heart.

Renin concentrations are inversely associated with MC activity in patients with primary AI, as well as in the general population. Autonomous hypersecretion of aldosterone results in a higher cardiovascular morbidity and mortality compared with age- and sex-matched patients, with essential hypertension and the same degree of blood pressure elevation [14]. This increased risk persists even during medical treatment with MC antagonists by aldosterone receptor blockers, as long as plasma renin concentration remains suppressed [9]. Moreover, treatment with aldosterone antagonists in chronic heart failure is well established and known to reduce hospitalization and mortality, wherefore aldosterone antagonists are included in the therapeutic algorithm of recent clinical practice guidelines for heart failure therapy [19]. Aldosterone modulates different cardiovascular risk factors in many ways. Within the heart, aldosterone promotes cardiac fibrosis by stimulation of collagen and fibroblast proliferation, therefore contributing to left ventricular hypertrophy [20, 21]. Moreover, aldosterone not only affects arterial stiffness, vascular compliance, and relaxation, but also increases intravascular volume by sodium retention in the kidney, which all together contribute to an increased afterload and thereby to cardiac hypertrophy [22]. These mechanisms of aldosterone action are likely to explain the observed differences of left ventricular mass and function in our patients.

Of note, elevated renin is strongly correlated with increased concentration of AT2, which is well known for its adverse effects on vasculature and the myocardium, similar to aldosterone [11]. Therefore, it was speculated that an inappropriate reduction of fludrocortisone dose in patients with primary AI should be avoided to protect from the deleterious actions of AT2 [23]. This assumption cannot be confirmed by the results observed in our patients. The most likely explanation might be that in primary AI, increased RAS activity not only results in higher levels of AT2, but also increases concentrations of other potentially cardioprotective angiotensin metabolites like Ang1–7, what we observed in previous preliminary studies [13]. Ang1–7 is decreased in acute heart failure and might counteract AT2 by induction of vasodilation, therefore preventing the development of left ventricular hypertrophy [12, 24]. Thus, we suppose that the observed rise in RAS activity in primary AI, which closely correlates with plasma renin concentration [13], results in a well-balanced increase of various angiotensin metabolites with protective, as well as harmful effects on cardiovascular risk factors.

Only limited data exist on heart function and the prevalence of heart failure in patients suffering from primary AI. Animal models nicely demonstrate the necessity of GC and MC replacement to maintain proper cardiac function in adrenalectomized rats [25]. In humans, older, cross-sectional studies in 22 patients with AI and long-term disease duration, heart failure was found in almost one-third of the patients [26]. This is in contrast to our study, in which systolic heart function was normal in all patients, despite a long disease duration of 19 ± 10 years. Systolic heart function was better in matched healthy controls compared with patients with primary AI. This difference was especially marked, when patients were divided according to their previous median renin concentrations, showing significantly worse ejection fraction in the group of Median-Renin_low_. Therefore, optimizing MC replacement therapy might help to prevent the development of heart failure in elderly patients with primary AI.

Clinical practice guidelines and expert comments recommend an upper normal or slightly elevated plasma renin concentration to aim for adequate MC replacement therapy [4, 14, 23, 27], which cannot be supported by our data. Due to the non-interventional, cross-sectional design of our study, we are not able to answer the question, if a reduction in daily fludrocortisone dose might be beneficial for heart function and morphology. However, our data clearly demonstrate that higher renin concentrations are associated with a favorable cardiac performance, i.e., better left ventricular systolic function and left ventricular smaller mass.

Of note, MC replacement plays an important role to maintain quality of life in patients with primary AI [28] and improves mood and cognition [29]. Evaluation of adequate MC therapy depends on clinical signs like blood pressure or salt craving, and biochemical markers, including sodium, potassium, and plasma renin concentration [23]. In our study, salt craving was two times more common, and also a postural drop in systolic blood pressure during the Schellong test was only observed in Actual-Renin_high._

A limitation of our study is the intra-individual variability of plasma renin concentration, mainly depending on daily salt and water intake, which was not standardized during the study days. To address this issue, Median-Renin concentrations of previous visits at the endocrine outpatients’ clinic were included for analysis to investigate the effects of RAS activity during a longer observation period. Of note, despite distinct changes in renin concentrations, 87.5% of all included patients stayed within their groups, i.e., Actual-Renin_low _= Median-Renin_low_/Actual-Renin_high_ = Median-Renin_high_.

With regard to GC replacement, large-cohort studies demonstrate an impact of the daily dose of hydrocortisone on conventional cardiovascular risk factors like hypertension, BMI, and waist circumference [5], whereas other studies were not able to confirm this association [30, 31]. When patients were divided into two groups according to their GC replacement therapy, glucose and lipid metabolism were not different in patients with >20 mg of hydrocortisone per day. This might be due to the small sample size of patients in each group, since routine laboratory parameters show high variation. However, also ectopic lipid content in the liver, as well as the ratio of subcutaneous to visceral adipose tissue, which are both sensitive markers for insulin sensitivity and energy homeostasis in smaller cohorts [32], were comparable.

Taken together, our results demonstrate that higher renin concentrations are associated with a favorable cardiac function and morphology in patients with primary AI. If a reduction in MC replacement therapy prevents the development of heart failure and reduces cardiovascular mortality should be evaluated in future prospective trials.
